# Steroidogenic factor-1 (SF-1) gene mutation as a frequent cause of primary amenorrhea in 46,XY female adolescents with low testosterone concentration

**DOI:** 10.1186/1477-7827-8-28

**Published:** 2010-03-19

**Authors:** Pascal Philibert, Elodie Leprieur, Delphine Zenaty, Elisabeth Thibaud, Michel Polak, Anne-Marie Frances, James Lespinasse, Isabelle Raingeard, Nadège Servant, Françoise Audran, Françoise Paris, Charles Sultan

**Affiliations:** 1 Service d'Hormonologie, Hôpital Lapeyronie, CHU Montpellier, and Université Montpellier 1, France; 2 Unité d'Endocrinologie et Gynécologie Pédiatrique, Hôpital Arnaud de Villeneuve, CHU Montpellier, and Université Montpellier 1, France; 3 Service de Pédiatrie, Hôpital Robert Debré, AP-HP, Paris, France; 4 Service d'Endocrinologie et Gynécologie de l'Enfant, Hôpital Necker-Enfants Malades, AP-HP, Paris, France; 5 Service de Génétique Médicale, Hôpital Intercommunal de Toulon-La Seyne sur Mer, Toulon, France; 6 Service de Génétique, Hôpital de Chambéry, Chambéry, France; 7 Service d'Endocrinologie, Hôpital Lapeyronie, CHU Montpellier, France

## Abstract

**Background:**

Primary amenorrhea due to 46,XY disorders of sex differentiation (DSD) is a frequent reason for consultation in endocrine and gynecology clinics. Among the genetic causes of low-testosterone primary amenorrhea due to 46,XY DSD, SRY gene is reported to be frequently involved, but other genes, such as SF1 and WT1, have never been studied for their prevalence.

**Methods:**

We directly sequenced SRY, SF1 and WT1 genes in 15 adolescent girls with primary amenorrhea, low testosterone concentration, and XY karyotype, to determine the prevalence of mutations. We also analyzed the LH receptor gene in patients with high LH and normal FSH concentrations.

**Results:**

Among the 15 adolescents with primary amenorrhea and low testosterone concentration, we identified two new SRY mutations, five new SF1 mutations and one new LH receptor gene mutation. Our study confirms the 10-15% prevalence of SRY mutations and shows the high prevalence (33%) of SF1 abnormalities in primary amenorrhea due to 46,XY DSD with low plasma testosterone concentration.

**Conclusions:**

The genetic analysis of low-testosterone primary amenorrhea is complex as several factors may be involved. This work underlines the need to systematically analyze the SF1 sequence in girls with primary amenorrhea due to 46,XY DSD and low testosterone, as well as in newborns with 46,XY DSD.

## Background

Adolescent primary amenorrhea is a frequent reason for consultation in pediatric and gynecological endocrine clinics. Primary amenorrhea may result from congenital abnormalities in gonadal or genital tract development or from a defect in the hypothalamic-pituitary-ovarian axis. Failure to menstruate by the age of 15 years requires investigation to determine the cause and establish a treatment plan. The standard investigation includes detailed clinical evaluation [1], endocrine assessment (gonadotropins, testosterone, AMH and inhibin B assays) and pelvic imaging. In addition, genetic exploration is crucial to classify the primary amenorrhea. Karyotyping, which discriminates normal from abnormal chromosomes (i.e., 45,X0 or 46,XY), is the first step. In the 46,XY disorders of sex differentiation (DSD) [2], primary amenorrhea may be caused by a genetic defect in fetal testis determination, failure of the fetal testis to produce testosterone, or androgen resistance [3]. The assessment of endocrine parameters thus often orients the exploration toward the most probable genetic cause [4]. For example, when plasma testosterone (pl-T) is low, an abnormality in the genes involved in fetal testis determination, such as *SRY*, *SF1*, *WT1*, *SOX9, DMRT, DHH, DAX1 *and *WNT4*, should be considered [5].

Here we describe a two-year experience of genetic exploration in adolescents with primary amenorrhea due to 46,XY DSD and low pl-T concentration. We specifically focused on *SRY, SF1 *and *WT1 *because these genes have previously been reported to be implicated in adolescents presenting with primary amenorrhea due to 46,XY DSD in association with low pl-T, but no other signs. Our aim was thus to assess the frequency of mutations in these genes in a cohort of 15 adolescents with this profile. We identified eight unreported mutations in these genes responsible for testis differentiation and development: two new mutations in *SRY *and five new mutations in *SF1*. Moreover, in a patient with a specific biological profile of elevated LH and normal FSH concentrations, we identified a new *LH receptor *mutation. Thus far, we have been unable to determine a genetic cause for the primary amenorrhea in the seven remaining subjects.

## Methods

### Patient cohorts

Over a two-year period (2007-2009), we studied 31 adolescent patients with primary amenorrhea due to 46,XY DSD who had been referred by collaborating centers to the gynecological unit of our pediatric endocrine clinic or to our genetics laboratory. All patients had a 46,XY karyotype. We were able to classify these adolescents with primary amenorrhea into two main groups. The first group was composed of 16 patients with high pl-T concentrations, whereas the second group comprised 15 patients with low pl-T (< 0.4 ng/ml) and elevated gonadotropins (Table 1). We focused our study on the second group. The phenotype of most of these patients was female and 5/15 of them presented with isolated clitoromegaly (Table 1).

**Table 1:** Clinical, endocrine and genetic features of the 15 adolescents with primary amenorrhea and low testosterone

	External genitalia	Tanner stages	Uterus	Gonads pathology	T (ng/ml)	FSH (UI/l)	LH (UI/l)	Mutation	Inheritance	Protein consequences
Patient 1	Normal female	B3P3	Present	Streak gonads	0.66	33	9.4	*SRY*: c.292T>C	Hemizygous	p.Trp98Arg
Patient 2	Normal female	ND	Present	ND	< 0.1	13	2	*SRY*: c.319insA	Hemizygous	p.Trp107Met*fsX21*
Patient 3	Normal female	ND	ND	ND	< 0.1	ND	ND	*SF1*: c.1A>G	Heterozygous	p.Met1Val (probably p.0)
Patient 4	Clitoromegaly	ND	ND	Gonads in labia	< 0.1	ND	ND	*SF1*: c.116G>C	Heterozygous	p.Arg39Pro
Patient 5	Clitoromegaly	ND	ND	Testicular tissue/inguinal region	0.7	ND	ND	*SF1*: c.151delG	Heterozygous	p.Glu51Arg*fsX23*
Patient 6	Clitoromegaly + Virilization	ND	ND	ND	2,2 (post HCG)	ND	ND	*SF1*: c.369insC	Heterozygous	p.Pro124Pro*fsX24*
Patient 7	Normal female	B1A2P5	Present	ND	0.3	40	18	*SF1*: c.1138G>T	Heterozygous	p.Asp380Tyr
Patient 8	Normal female	B1A3P3	ND	Presence of Sertoli cells	0.33	8.7	11.2	*LHCGR*: c.1395G>A	Homozygous	p.Trp465X
Patient 9	Normal female	ND	Absent	Streak gonads	0.35	102	28	None	-	-
Patient 10	Normal female	B1P1	ND	ND	0.38	135	42	None	-	-
Patient 11	Clitoromegaly + Hirsutism	ND	Present	Gonadoblastoma	0.3	ND	ND	None	-	-
Patient 12	Normal female	ND	Present	Streak gonads	< 0.1	78	46	None	-	-
Patient 13	Normal female	ND	ND	Gonadal dysgenesis	< 0.1	59	45.8	None	-	-
Patient 14	clitoris and labia agenesis	B2P4	Present	Left dysgerminoma, Right fibrous structure (few tubules)	0.14	71.7	18.6	None	-	-
Patient 15	Clitoromegaly	B1P4	Absent	ND	0.3	56.3	29.5	None	-	-

Normal ranges: basal T, 0.35-0.9 ng/ml; FSH, 1-12 UI/l; LH, 1-5 UI/l. Conversion to SI: T ng/ml × 3,47 for nmol/l.

ND: Not Determined

### Mutation analysis

With the informed consent of the patients and/or their parents, DNA was extracted from peripheral blood leukocytes. The study was approved by the institutional review boards of all collaborating hospitals. The entire coding region and splice sites of the *SRY*, *NR5A1 *(*SF1*) and Wilm's tumor (*WT1*) genes were PCR-amplified using previously described primers and conditions [6-8] and sequenced directly using BigDye terminator v1.1 (Applied Biosystems) and an ABI Prism 3130 genetic analyzer (Applied Biosystems).

### Predicted SRY and SF1 mutation effects

Amino acid substitutions were studied *in silico *to predict the effects. We performed the *in silico *analysis using two software packages, PolyPhen [9] and SIFT [10]. The PolyPhen algorithm is able to predict the functional effects of amino acid changes by considering evolutionary conservation, physicochemical differences, and the proximity of the substitution to predicted functional domains and/or structural features. The SIFT algorithm predicts the functional importance of amino acid substitutions based on the alignment of orthologous and/or paralogous protein sequences. Scores lower than 0.05 suggest the potential pathogenicity of mutations. The original protein sequences were obtained from the Ensembl and UniProt/Swiss-Prot databases.

## Results

### Mutation analysis

**Figure 1:**
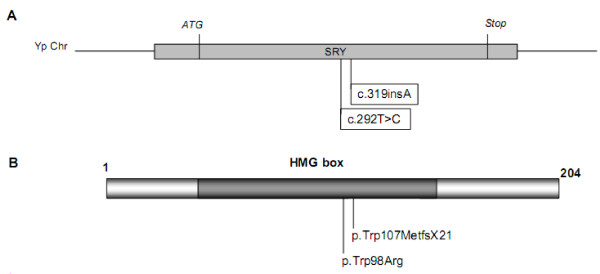
**Representation of SRY gene (A) and protein (B) showing the localization of mutations identified in adolescents with primary amenorrhea due to 46,XY DSD**.

Direct sequencing of *SRY *revealed two new mutations in patients 1 and 2 (Table 1). Patient 1 showed a variant, c.292T>C, leading to the amino acid p.Trp98Arg substitution in a highly conserved HMG domain (Figure 1). This variant was absent in 100 control chromosomes. In patient 2, we identified a new nucleotide insertion in *SRY *(c.319insA). This insertion led to a premature stop codon 21 amino acids after the mutated codon in the DNA binding domain, which doubtlessly abolished the *SRY *function.

**Figure 2:**
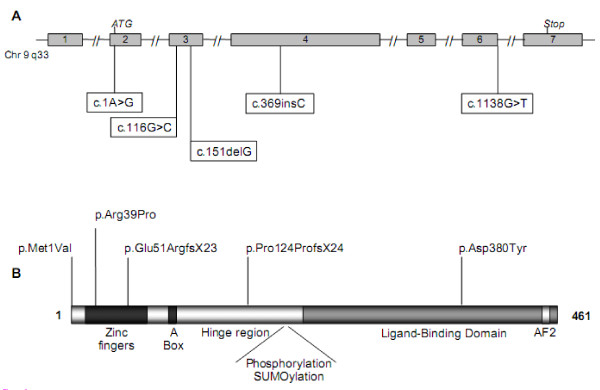
**Representation of SF1 gene (A) and protein (B) showing the localization of mutations identified in adolescents with primary amenorrhea due to 46,XY DSD**.

When the *SRY *sequence was normal, we systematically studied the *SF1 *and *WT1 *sequences. We identified five new heterozygous *SF1 *mutations in patients 3, 4, 5, 6 and 7 (Table 1). Among them, three were nucleotide substitutions: c.1A>G (p.Met1Val) for patient 3, c.116G>C (p.Arg39Pro) for patient 4, and c.1138G>T (p.Asp380Tyr) for patient 7. The two other *SF1 *mutations were a deletion, c.151delG, for patient 5 and an insertion, c.369insC, for patient 6 (Figure 2). These mutations led to a frame shift with a premature stop codon 23 and 24 amino acids later, respectively. Proteins resulting from these mutations are predicted to have null activity. No mutation of *WT1 *was identified in our group.

**Figure 3:**
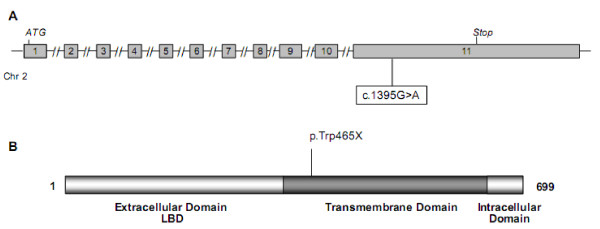
**Representation of LHCGR gene (A) and protein (B) showing the localization of mutations identified in adolescents with primary amenorrhea due to 46,XY DSD**.

As patient 8 had elevated LH and normal FSH concentrations (Table 1), we performed *LHCGR *analysis. The direct sequencing of this gene revealed a new homozygous LH receptor mutation, c.1395G>A (p.Trp465X), leading to a stop codon that causes an aberrant transmembrane domain (Figure 3). As most of the transmembrane domain was missing, it was certain that the LH receptor was non-functional.

### Prediction of missense mutation effects

We used two software packages to predict the functional effects of the amino acid substitutions. First, we performed testing with PolyPhen [11]. Except for the missense mutation affecting the initiating codon, all missense substitutions were classed as "probably damaging".

The same assessment was performed with SIFT [12]. Similar to the PolyPhen method, the SIFT score for these *SRY *and *SF1 *substitutions was 0.00, except for p.Met1Val, which cannot be predicted by this software. This score placed the missense mutations in the "Affects protein function" class and confirmed the evaluation by the PolyPhen algorithm.

The other mutations were an insertion and a deletion, and the prediction software was unable to assess their deleterious effects. However, these mutations lead rapidly to premature stop codons and truncated proteins, with probably altered function. For the LH receptor, prediction of the nonsense mutation effects was not necessary since the amino acid substitution leads to a stop codon within the transmembrane domain and results in a protein with no intracellular domain.

## Discussion

Primary amenorrhea is a frequent reason for consultation in pediatric endocrine and gynecology clinics. Three types of primary amenorrhea can be observed: (1) 46,XX amenorrhea with high FSH concentration, which usually corresponds to a reduction in the number of primary follicles, accelerated follicular atresia, or follicular dysfunction; (2) 45,X0 amenorrhea associated with Turner syndrome; and (3) amenorrhea due to 46,XY DSD. This last can be further classified into two subtypes. The first is defined by primary amenorrhea with normal or high testosterone concentration, suggesting androgen insensitivity syndrome or possibly 5-alpha reductase type 2 deficiency. The second is defined by primary amenorrhea with low testosterone concentration. The etiologies are multiple and testis determination genes such as *SRY*, *SF1 *or *WT1 *should be explored.

In our 31 adolescents with primary amenorrhea due to 46,XY DSD, 16 showed high testosterone concentrations and, as expected, *AR *gene mutation was a major cause among those who presented with breast development (B5) contrasting with an absence of pubic hair (P1) (data not presented). For the group with low testosterone concentration and considered as 46,XY gonadal dysgenesis [13], we systematically analyzed *SRY*, *SF1 *and *WT1 *genes, beginning with *SRY*. Gene analysis identified two new *SRY *mutations (2/15). This frequency (13.3%) is similar to that usually reported in the literature, which varies from 10 to 15% [14], and suggests that our cohort was representative of the population of adolescents with primary amenorrhea due to 46,XY DSD and low pl-T.

Patients without *SRY *gene mutations were analyzed for defects in *WT1 *or *SF1*. *WT1 *is normally associated with Frasier or Denys-Drash syndrome. In our experience with this gene analysis, we have identified a mutation three times in patients with no sign of kidney abnormality or cancer (unpublished). However, no cases of *WT1 *abnormality were noted in the present study group.

In contrast, we identified five new *SF1 *mutations in this cohort (5/15), which amounts to one third of the cases of primary amenorrhea due to 46,XY DSD with low pl-T level and 5/31 of the cases of primary amenorrhea due to 46,XY DSD. Among these new mutations, three nucleotide substitutions, one insertion and one deletion were identified.

Although *in vitro *studies are needed to demonstrate the implication of these mutations, two of our *SF1 *mutations and the *SRY *insertion certainly abolished activity since an insertion or deletion creates a frame shift and premature stop codon. The p.M1V *SF1 *mutant probably abolished the transcriptional initiation. An alternative initiation codon is located downstream at codon 78, after the DNA-binding domain, and probably altered the SF1 function. A similar mutation in the initiation codon (p.Met1Ile) was reported in a girl with hypertrophic clitoris, which confirms the impact of this abnormality [15]. These three cases are similar to the cases of haploinsufficiency reported by our group and others [16-18] in patients with complete sex reversal. For the two other *SF1 *missense mutations and the *SRY *substitution reported here, the functional effects predicted by the two types of software were concordant, with the same conclusion of affected protein mutants. These predictions agreed with the phenotype and hormonal data observed in our patients, and we can conclude that these mutations were probably the cause of the phenotype and the biological abnormalities.

We analyzed the LH receptor gene in one of the girls of our cohort because of high LH contrasting with normal FSH concentration [19]. We were thus able to identify a previously unreported *LHCGR *mutation leading to an inactive truncated LH receptor.

For the seven primary amenorrhea adolescents with low pl-T concentrations and no mutation in any of the studied genes, it is probable that one of the other genes implicated in DSD was mutated. The absence of associated signs was a supplementary difficulty for the orientation of genetic exploration, but further gene analysis is warranted to identify the etiology of the DSD observed in these patients. Any one of several genes could be involved, such as *SOX9*, *DMRT*, or *DHH*, as well as duplications of *DAX1 *or *WNT4*. However, these genes are not often found to be mutated, especially without specific associated signs.

## Conclusions

To conclude, the genetic analysis of low-testosterone primary amenorrhea due to 46,XY DSD is complex since several factors may be involved, including SRY, SF1, WT1 and LH receptor. We confirmed that *SRY *was mutated in about 10 to 15% of the cases. More interestingly, we identified new *SF1 *mutations in five of our 15 patients. As this amounts to one third of our cohort, we suggest that the *SF1 *sequence should be systematically analyzed in girls with primary amenorrhea due to 46,XY DSD and low testosterone concentration, as well as in newborns with 46,XY DSD.

## Competing interests

The authors declare that they have no competing interests.

## Authors' contributions

PP participated in the design of the study and the molecular genetic analyses, carried out the sequence alignment and the *in silico *analyses, and drafted the manuscript. El collected the clinical data and participated in patients follow-up. DZ, ET, MP, AMF, JL and IR all managed patients. NS carried out a part of the molecular genetic study. FA carried out the other part of the molecular genetic study. FP managed patients and participated in patients follow-up. CS conceived the study, participated in its coordination, and helped to draft the manuscript. All authors read and approved the final manuscript.
